# Unilateral and Bilateral PNIF in Quality Control of Nasal Septal Surgery

**DOI:** 10.1155/2018/7846843

**Published:** 2018-10-16

**Authors:** Liv Kari Døsen, Knut Kvinnesland, Magnus TarAngen, Olga Shiryaeva, Caryl Gay, Rolf Haye

**Affiliations:** ^1^Department of Otorhinolaryngology, Lovisenberg Diaconal Hospital, Oslo, Norway; ^2^Department of Quality, Lovisenberg Diaconal Hospital, Oslo, Norway; ^3^Institute of Clinical Medicine, Faculty of Medicine, University of Oslo, Oslo, Norway

## Abstract

The aim of this study was to explore the usefulness of unilateral, combined unilateral (left + right), and bilateral peak nasal inspiratory flow (PNIF) measurements in assessing the results of nasal septal surgery. Nasal obstruction was recorded subjectively and objectively before and 4 months after nasal septoplasty using a visual analogue scale (VAS) and a PNIF meter. Nasal septoplasty (58 patients) and septoplasty with turbinoplasty (68 patients) were performed on 126 patients (85 males; 41 females) with a mean age of 32.8 years. The results showed a significant improvement in VAS scores, as well as unilateral, combined unilateral, and bilateral PNIF values after both septoplasty and septoplasty with turbinoplasty. Septoplasty with turbinoplasty showed better improvement in VAS and PNIF scores than septoplasty alone and this was significant for bilateral PNIF scores. The best unilateral pre- and postoperative correlations between VAS and PNIF measurements were found using the lower of the two unilateral PNIF scores, irrespective of side. In the total material, VAS/PNIF correlations were mostly significant, but weak (all r<0.30). We found VAS and PNIF to be useful instruments in reporting results of surgery. The weak correlations between VAS and PNIF measurements suggest that these subjective and objective instruments may target different aspects of nasal obstruction.

## 1. Introduction

Nasal septal deviation is a common cause of nasal obstruction that can be treated surgically. The decision to operate is usually made by the surgeon on clinical grounds alone without using objective measurements. The results of surgery are not always satisfactory and preoperative objective measurements might therefore improve the selection of patients for surgery. Rhinomanometry is regarded as the gold standard in objective measurements of nasal obstruction, but it is relatively expensive and time consuming and requires experience. In contrast, a peak nasal inspiratory flow (PNIF) meter is inexpensive and measurements are quick and easy to perform [1]. Bilateral measurements have been used for many years to assess the results of nasal allergy treatment. Unilateral measurements, while not widely used clinically, have been included in some studies of nasal obstruction [2]. The use of PNIF measurements has not been approved for assessing the results of treatment of unilateral nasal obstruction [3]. Thus, further studies of PNIF measurements for this purpose should be explored.

The results of surgery are usually assessed by questionnaires. At our hospital, we routinely perform quality control of nasal septal surgery [4] using mailed nasal surgical questionnaires (NSQ), which include visual analogue scales (VAS) for obstruction. Some of our surgeons recall patients for a clinical examination four months after surgery. At this visit, patients complete the postoperative version of the NSQ, the PNIF measurements are performed, and the surgeon's clinical findings are recorded.

In addition to subjective questionnaires, objective measurements may be of great value in selecting patients for therapy and for comparing the results of different surgical techniques. Thus, the primary purpose of this study was to assess the clinical value of unilateral, combined unilateral (i.e., left + right), and bilateral PNIF measurements in the evaluation of prospectively recorded results of nasal septoplasty. Since perceived nasal obstruction might only be related to the unilateral PNIF score for the side with greater obstruction, a secondary objective was to examine the association between VAS scores and unilateral PNIF scores on the side with the lower pre- and postoperative values, irrespective of the side on which this was observed.

## 2. Materials and Methods

This study was approved by the Ethics Committee of Lovisenberg Diaconal Hospital in a letter dated 28.11.2014. The Regional Ethical Committee of South-Eastern Norway Section C (2016/2301/REK sør-øst C) decided that PNIF measurements in quality control studies are exempt from regional ethical approval. Patients with nasal septal deviation treated with nasal septoplasty with or without turbinoplasty by two surgeons from September 2014 to February 2016 were included. The turbinoplasty was performed as a submucous partial bony resection of the inferior turbinate. It was only done unilaterally. The same surgeons performed both the initial clinical examination and the follow-up after three and a half to five months. Patients were excluded if they had any other nasal or sinus disease except nasal allergy, were undergoing any other concomitant nasal or sinus surgery, or were unable to comply with the PNIF measuring technique.

**Figure 1:**
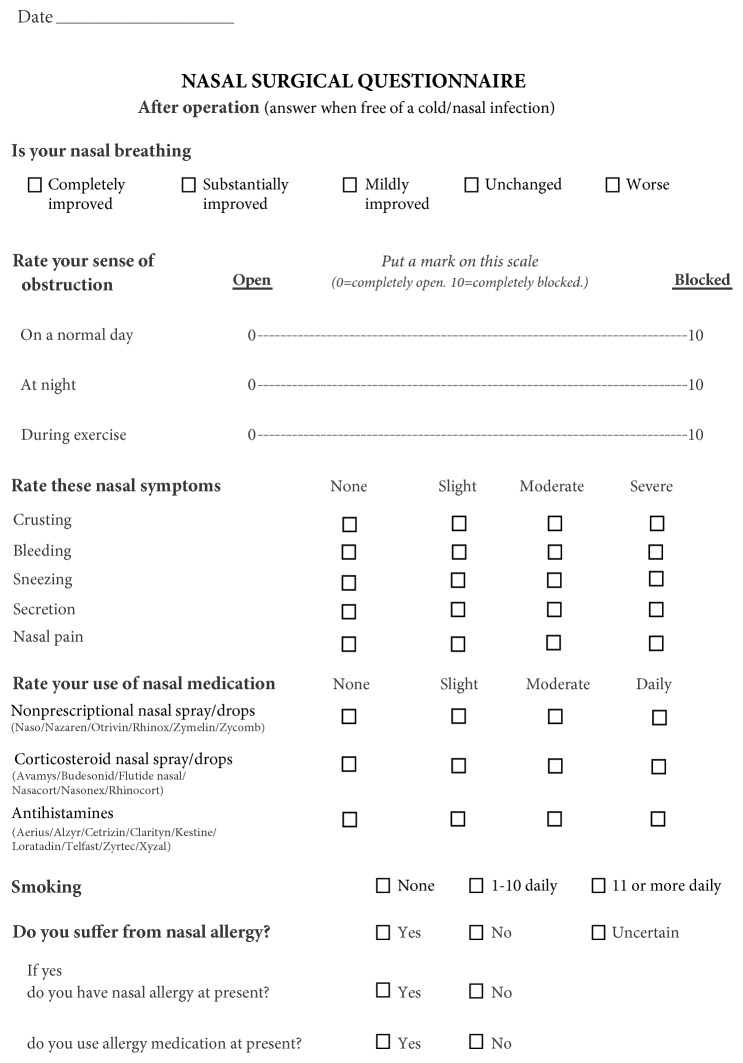
Nasal Surgical Questionnaire (postoperative version).

On the day of surgery, patients completed the preoperative version of the nasal surgical questionnaire (NSQ). Both the pre- and postoperative (Figure 1) versions of the NSQ have separate visual analogue scales (VAS) for obstruction during the day, at night and during exercise. The scales are 10 cm long with markings of* 0 = completely open* and* 10 = completely obstructed* at either end. The patients are asked to mark their sense of obstruction on this scale. Scores are the distance between the mark and the left end of the line (measured in mm) and can range from 0 to 100. The VAS scores are measured and recorded manually, whereas answers to the rest of the questions, which are marked in boxes, are recorded automatically by scanning. Five other nasal symptoms (crusting, bleeding, sneezing, secretion, and nasal pain) and use of 3 nasal medications (vasoactive drugs, topical steroids, and antihistamines) are rated on 4-point Likert scales (1= none, 2= slight, 3= moderate, and 4= severe/daily). In addition, items about smoking habits and self-reported allergy are included. The postoperative NSQ is supplemented with the following 5-point retrospective rating of perceived improvement: Is your nasal breathing completely, substantially, mildly or not improved, or has it deteriorated? Patients are asked to answer the questionnaire based on a normal day without nasal infection.

Prior to the clinical examination (which included nasal endoscopy), peak nasal inspiratory flow (PNIF) measurements were performed using a Youlten PNIF meter (Clement Clark International). The patient was seated, rested for 15 minutes and cautiously blew his nose. The airflow was first measured bilaterally, then on the right, and finally on the left side. Three readings were recorded for each of the measurements, but only the best was used for the evaluation. We used nonpermeable silk tape to close the nontested nasal nostril taking care not to deform the shape of the nostril. All measurements were performed in the morning. As the PNIF measurements were performed in the morning, we used the VAS scores during the day for comparison. All PNIF scores are in L/minute.

Immediately after surgery, the surgeon recorded in the surgical log the type of surgery performed and the following clinical data: side (right, left, or bilateral) and grade of septal deviation (slight = less than the half width of the nasal cavity, moderate = half the width of the nasal cavity, or severe = subtotal to total obstruction), hypertrophy of inferior concha (skeletal, soft tissue, or both), and side (right, left, or bilateral) and grade of nasal crusting (slight, moderate, or severe).

Three and a half to five months postoperatively the patients were recalled for a clinical examination, which again took place during the morning. The patient first completed the postoperative version of the NSQ, followed by the PNIF measurements. Finally, the surgeon examined and recorded the same clinical data as was done preoperatively and reported on any signs of infection or presence of allergy or asthma.

### 2.1. Statistical Analyses

Continuous data were presented as means with standard deviations (SD) and categorical variables as numbers (percent). Group comparisons were performed with independent-sample t*-*tests. Paired-sample t-tests were used to analyse pre- to postoperative changes in VAS and PNIF scores. Pearson correlation analyses were performed to determine the degree of association between VAS and PNIF scores. Multivariable linear regression analyses were applied to estimate whether the results of correlation analyses were still reliable when controlling for the effects of other relevant variables. All tests were two-sided; p-values <0.05 were considered statistically significant. All analyses were conducted using SPSS for Windows, version 24.0 (IBM Corp, Armonk, NY).

## 3. Results

Of the 128 patients entered into the study, 126 (85 males and 41 females) were included in the analysis. One patient did not return for follow-up and another patient was pregnant at the time of the follow-up and therefore excluded. The mean age was 32.8 ± 11.8 years. There were eleven daily smokers, ten patients with asthma, all using medication when needed, and 45 (35.7 %) reported having had nasal allergic symptoms at one time or another during the past year. Septoplasty with turbinoplasty was performed on 68 of the patients, while septoplasty alone was performed on the remaining 58 patients. Twelve patients were unable to attend the appointment scheduled for four months after surgery, and their clinical examination therefore took place later (five at six months, three at seven months, three at eight months, and one at twelve months). In ten patients, postoperative bilateral PNIF readings failed to be recorded.

The pre- and postoperative and change scores for the VAS and unilateral, combined unilateral (i.e., left + right), and bilateral PNIF for septoplasty, septoplasty with turbinoplasty, and total sample are shown in Table 1. There were statistically significant improvements in VAS and all PNIF scores after surgery in all three groups. Septoplasty with turbinoplasty showed better improvement in VAS and all PNIF scores. For bilateral PNIF ratings this was significant (p=0.01).

As shown in Table 2, there were no statistically significant differences in mean preoperative, postoperative, or change in PNIF scores between males and females (p=0.79), between smokers and nonsmokers (p=0.31), between older (≥35 years) and younger patients (p=0.48), patients with asthma and without (p=0.15), or patients reporting and not reporting allergic rhinitis (p=0.78). The preoperative VAS score was lower in allergic than in nonallergic patients (p=0.027) and the postoperative mean VAS score was lower in older than younger patients (p=0.027). In light of these differences, age and nasal allergy were included as covariates in the multiple regression analyses.

For each of the pre- and postoperative measurements in the total material, VAS scores were significantly correlated with PNIF scores (p<0.01), except for the preoperative PNIF measure on the left side (Table 3). In terms of the change between pre- and postoperative scores, only the right and bilateral PNIF change scores were correlated with VAS change scores and less strongly than for the pre- and postoperative correlations.

Nasal medication was used by some of the patients, although very few used the same type of medication both pre- and postoperatively. The number of patients using nasal medication on a daily basis at each assessment and at both assessments is presented in Table 4. None of the patients using topical steroids preoperatively were the same as those who used them postoperatively. Only two patients used more than one medication. We compared the VAS and PNIF scores for patients using each medication (decongestants, topical steroids, and antihistamines) on a daily basis to those not using medication on a daily basis. There were no significant differences in VAS and PNIF scores, regardless of whether the patients were using each of these medications.

The secondary objective was to assess the relationship between VAS scores and unilateral PNIF scores for whichever side had more obstruction. There was a significant correlation between the VAS and unilateral PNIF scores on the more obstructed side both preoperatively (r=0.322, p<0.001) and postoperatively (r=0.280, p=0.02). However, the pre- to postoperative change in PNIF scores on whichever side was more obstructed was not correlated with the change in VAS scores (r= 0.119, p=0.185).

To examine whether the observed correlations between the VAS and PNIF parameters were influenced by the effects of age and nasal allergy, multiple regression analyses were performed. The results of these adjusted analyses indicated that while the direction of associations was the same, the strength of the VAS and PNIF associations was somewhat attenuated by controlling for these confounding factors.

## 4. Discussion 

In this prospective study of the results of nasal septal surgery, we found statistically significant improvement in both subjective (VAS) and objective (PNIF) measurements following nasal surgery whether septoplasty alone or septoplasty with turbinoplasty was performed. Septoplasty with turbinoplasty showed more improvement in all measurements than septoplasty alone. We believe this may be due to the surgical removal of more of the skeletal structures in the nose when turbinoplasty is also performed.

Looking at the total surgical cohort, we found a significant correlation between the VAS scores and the unilateral and bilateral PNIF recordings both pre- and postoperatively, except for the preoperative measure on the left side. One explanation for the inconsistent unilateral findings may be related to the side toward which the septum was deviated. Preoperatively, most septal deviations were to the right, and thus, there should be lower PNIF values—and stronger associations with VAS—on the right than the left side, as was observed in this study. This explanation is also supported by the significant correlation found between VAS and the PNIF scores on the more obstructed side both pre- and postoperatively. Postoperatively, there were only a few minor deviations equally distributed to each side. A difference in PNIF values between the sides would therefore not be expected, nor would a difference in their associations with the VAS scores, which is consistent with our findings.

The improvement in subjective scores after nasal surgery in our study is similar to other studies of nasal surgery [5–7] both for septoplasty alone and for septoplasty with turbinoplasty. The improvement in bilateral PNIF values after septoplasty is similar to one report [6] but lower than in another [5]. The degree of improvement in bilateral PNIF ratings in septoplasty with turbinoplasty was similar to several other studies [8, 9] and also to studies on rhinoplasty [7, 10]. In our study, the overall mean preoperative bilateral PNIF score was lower than the reference value of 120 L/min considered discriminative between obstructive and normal values [11], while the mean postoperative PNIF value was well above this reference.

Moderate correlations between subjective and bilateral PNIF pre- and postoperative scores were found in some studies of the results of nasal surgery [5, 6, 8, 10], whereas in three others, no correlation was found [7, 12, 13]. In studies comparing normal persons versus patients with nasal obstruction [1, 14] and treatment of allergic rhinitis [15], moderate correlations were found between subjective and PNIF scores, while in others, this was not seen [16–18]. In our study, there were correlations between VAS and the bilateral and combined unilateral PNIF scores for both pre- and postoperative measurements in the total surgical material. The discrepancies between studies are difficult to explain.

There are relatively few studies comparing the change in VAS and PNIF scores after treatment. Two studies found significant correlations in the change from pre- to postoperative treatment scores [10, 15], whereas one [13] that found no correlation at the pre- and postoperative response was able to find a week correlation in the change of scores after surgery. Our study also confirms these findings that there are correlations between the VAS and bilateral PNIF pre- to postoperative change scores.

Our preoperative unilateral PNIF values were lower than in a group of patients with nasal obstruction due to a composite of diagnoses [1]. Our postoperative values were slightly lower than in normal persons [2] which may not be surprising.

The VAS and unilateral PNIF change scores were only correlated on the right side. There was no correlation between change in scores on the VAS and PNIF scores on the more obstructed side, even though they were correlated both pre- and postoperatively. It raises the question about the usefulness of unilateral PNIF measurements. The subjective sense of obstruction is probably more complex than objective measurements can provide.

The unilateral Nasal Obstruction Symptom Evaluation (NOSE) has been shown to be correlated with unilateral PNIF recordings [19]. Our patients were not asked to provide unilateral VAS ratings, and thus, we are unable to determine whether they would have better associations with unilateral PNIF ratings. To assess the overall results of surgery, however, it is likely that it is the overall sense of daytime nasal obstruction that matters most to the patient.

Nasal medications influence the nasal mucous membrane. Nasal decongestants cause a detumescence of the mucosa. Daily use, however, causes rhinitis with a swelling of the mucosa. Thus, this condition may have caused a preoperative increase in nasal obstruction. However, we were unable to verify this in our patients, as there were no differences on VAS or PNIF measures based on medication use. Topical nasal steroids were used by allergic patients to relieve symptoms. However, as no patient used them daily both pre- and postoperatively, we presume that they were used only when the patient needed them for their symptoms, thereby reducing the effect of their allergy on the nasal mucous membrane. Antihistamines were also used by allergic patients to reduce symptoms. The VAS scores in allergic patients were not higher than for nonallergic patients. We believe that the use of topical steroids and antihistamines helped reduce the effect of allergy on the nasal mucous membrane. Unfortunately, few studies report on the use of nasal medication, so it is difficult to compare the effects of medication with other studies.

Compared to our study, the ratio of females to males was higher in two studies [6, 9] and similar in four others [7, 10, 12, 20]. In one study [5], the gender ratio was not reported. The mean age was similar across all studies. Therefore, our sample seems to be demographically similar to other studies and likely representative of patients treated with septoplasty with or without turbinoplasty.

Two studies reported different effects of smoking on PNIF values [14, 21]. Asthma reduced PNIF values in one [22] but not in another study [14]. In our study, we did not find any significant difference in PNIF values in smokers, asthmatics, or those with allergy. Most of our patients were operated and clinically examined outside of their pollen seasons. Patients with asthma used antiasthmatic medication when needed.

The subjective obstruction scores and PNIF data in our and other studies are so similar that confounder items may only have a minor influence. To minimise any disturbance of the nasal airways, our PNIF measurements were performed in the morning before the clinical examination.

Strengths of this study are that it is prospective and includes patients operated by only two surgeons. A limitation is that more information on the relationship between VAS and unilateral PNIF recordings may have been found by including unilateral VAS ratings. We have not performed PNIF after decongestion and we recommend that this should be done in future studies. We only performed unilateral turbinoplasties. In future studies it should be declared whether the turbinoplasties were uni- or bilateral and any difference in results between them should be discussed.

## 5. Conclusion

Our findings of objective and subjective measurements are in line with most other studies. The subjective and objective improvements were significant showing that the subjective and objective instruments are valid. Septoplasty with turbinoplasty showed better improvement particularly with bilateral PNIF scores, indicating better improvement in breathing, than septoplasty alone. The unilateral correlations indicate that the subjective sense of obstruction is best related to whichever side had lower PNIF scores (i.e., more obstruction). This correlation, however, was not evident with the improvement scores. Bilateral PNIF measures overall had the strongest associations with VAS scores in terms of change after surgery. We expected stronger correlations between subjective and objective change scores. The diverging findings of improvement in the different unilateral recordings compared to the subjective ones should be further explored. The VAS and PNIF measurements were from two different instruments. The correlations between them were weak, suggesting that they may be measuring different aspects of nasal obstruction.

## Figures and Tables

**Table 1:** PNIF and VAS preoperative, postoperative, and change scores for septoplasty, septoplasty with turbinoplasty, and the total sample.

Measure	N	Preoperative	Postoperative	Change*∗*	p value
***Septoplasty only***					

VAS day	58	62.34 (22.15)	21.40 (19.77)	40.95 (26.78)	<0.001

PNIF right	58	63.88 (37.10)	90.10 (32.42)	26.21 (33.03)	<0.001

PNIF left	58	68.02 (37.71)	87.93 (35.85)	19.91 (32.99)	<0.001

PNIF right+left	58	131.90 (50.98)	178.02 (62.41)	46.12 (43.33)	<0.001

PNIF bilateral	53*∗∗*	107.92 (35.98)	132.74 (37.65)	24.81 (28.10)	<0.001

***Septoplasty with turbinoplasty***					

VAS day	68	63.18(22.15)	20.15( (17.64)	43.03 (23.00)	<0.001

PNIF right	68	57.72 (32.79)	90.66 (40.32)	32.94 (36.45)	<0.001

PNIF left	68	62.35 (29.53)	88.31 (37.495)	25.96 (26.15)	<0.001

PNIF right+left	68	120.07 (44.08)	178.97 (70.34)	58.90 (49.00)	<0.001

PNIF bilateral	63*∗∗*	104.21 (39.65)	144.60 (47.660)	40.40 (34.65)	<0.001

***Total sample (Septoplasty and Septoplasty with turbinoplasty combined)***					

VAS day	126	62.37 (21.64)	20.61 (18.64)	41.75 (25.29)	<0.001

PNIF right	126	61.83 (33.92)	91.03 (37.77)	29.21 (34.35)	<0.001

PNIF left	126	65.71 (32.95)	88.41 (36.78)	22.70 (29.52)	<0.001

PNIF right+left	126	127.54 (46.85)	179.44 (67.21)	51.90 (46.91)	<0.001

PNIF bilateral	116*∗∗*	106.21 (38.11)	139.44 (43.84)	33.23 (32.61)	<0.001

*∗*VAS change = Preoperative score – Postoperative score; PNIF Change = Postoperative score – Preoperative score.

*∗∗*A total of 10 patients (5 for each type of surgery) did not have postoperative bilateral PNIF measurements, resulting in lower sample sizes for these analyses.

**Table 2:** Preoperative and postoperative VAS and PNIF scores by patient characteristics.

Characteristic	n	VAS		PNIF	
		Preoperative	Postoperative	Preoperative	Postoperative
Male	85	63.1	19.8	131.1	187.6

Female	41	60.8	22.2	120.1	162.4

Age ≥35 years	46	63.3	16.4	127.3	180.5

Age <35 years	80	61.8	23.0*∗*	127.79	178.8

Asthmatic	10	69.5	25.2	147.0	199.0

Nonasthmatic	116	61.8	20.2	125.8	177.8

Allergic	45	57.6*∗*	20.6	126.7	174.6

Nonallergic	81	65.0	20.6	128.0	182.1

Smoker	11	66.2	25.3	125.1	171.4

Nonsmoker	115	62.0	20.2	127.8	180.2

*∗*p<.05.

**Table 3:** Correlations between VAS and PNIF scores pre- and postoperatively and the change from pre- to postoperative measurements.

Correlations with VAS:	Preoperative		Postoperative		Change	
	r	p-value	r	p-value	r	p-value
PNIF right	-0.255	0.004	-0.264	0.003	0.183	0.041

PNIF left	-0.098	0.273	-0.266	0.003	0.030	0.743

PNIF right+left	-0.254	0.004	-0.291	0.001	0.152	0.089

PNIF bilateral	-0.276	0.002	-0.282	0.002	0.207	0.026

**Table 4:** Number of patients using nasal medication daily at either the pre- or postoperative assessments or at both.

	Only Preoperative	Only Postoperative	Pre- and postoperative
Decongestants	9 (7.0%)	2 (1.6%)	1 (0.8%)

Topical steroids	12 (9.5%)	8 (6.3%)	0 (0.0%)

Antihistamines	6 (4.8%)	7 (5.6%)	5 (4.0%)

## Data Availability

Data may be obtained from Olga Shiryaeva.
